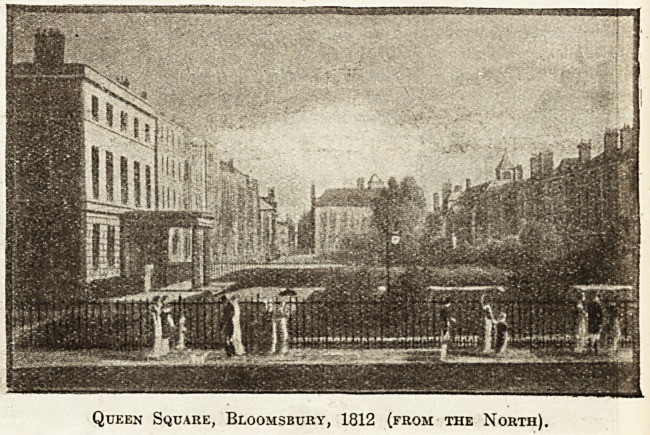# Queen Square

**Published:** 1921-04-02

**Authors:** 


### HOSPITAL NEIGHBOURHOODS.

### I. Queen Square.

Entrance to the National Hospital.

We are able to ascertain from the records of the Grey Friars', or Franciscan, Convent, Newgate Street (the buildings of which were afterwards occupied by Christ's Hospital), that the water supply of this religious house was brought from a spot in what is now Queen Square, Bloomsbury, which in this way, seven or eight hundred years ago, had an institutional connection. The writer saw the springhead shortly before the war, but it has since been built over by a Turkish-bath proprietor.

It was not, however, until the nineteenth century that the Square became a hospital neighbourhood; in that century four hospitals commenced their work within the three sides that are built upon—the fourth is a garden—and another hospital is hard by. Early in the century there were forty-five fair-sized houses in the Square; to-day fifteen of these have given place to hospital buildings, and another four are used for hospital purposes; the site of two more is occupied by a nurses' home, and of another four by the Examination Hall of the Royal Colleges of Physicians and Surgeons. But we must hark back to earlier times.

About 1700, "divers gentlemen," at the extreme boundary of the parish of St Andrew's, Holborn, proposed the erection of a chapel for the convenient performance of their religious duties. That chapel —now the church of St. George the Martyr—and one or two of the houses on the south side were the beginnings of a square which had been designed but not built. Soon afterwards houses of a substantial type filled three sides—the fourth being left open to afford a view of the country in the direction of Highgate.

From that time onward the houses became the residences of many famous, fashionable, and titled people. The then Bishops of Carlisle, Chester, and Chichester lived there in 1728, and Jonathan Richardson the elder, the distinguished portrait-painter and writer on art, died there in 1745. Many other names of note occur in a pamphlet by Mr. Philip Norman, F.S.A., to whom we owe much information. This able paper and our own notes supply the following mixed company:

The Alexandra Hospital in its First Home.

Edmond Hoyle, the authority on whist; Dr. Charles Burney, musician, author, and friend of Dr. Johnson; Fanny Burney, or Madame D'Arblay, daughter of Dr. Burney; Dr. John Campbell, man of letters; Miss Louise Twining, whose name was in most hospital subscription lists; William Morris, poet and printer; Mr. George Buckle, editor of *The Times*; Jerome K. Jerome; Robert Louis Stevenson; Mr. Clement Scott, dramatic critic; Nelly Farren, the wonderful actress; and many others. Captain Cook, the navigator, is known to have dined in one of the houses, Charles Churchill, the poet, taught in a ladies' school, and Dr. Johnson visited more than one of the residents many times. The largest house, Queen Square House, was for many years the residence of the legal family of Pollock. Until

Queen Square, Bloomsbury, 1787 (from the South).

**Hospital Neighbourhoods**—(*continued*).

the hospitals came and ousted them (there is but one private resident left) the above company and generations of citizens enjoyed the spacious rooms of the Georgian houses, with their beautifully moulded ceilings, mahogany doors, and handsome fireplaces. Several of these fine old buildings are still used for auxiliary purposes by the National Hospital for the Paralysed and Epileptic.

Two of the houses which the National Hospital displaced (Nos. 24 and 25) were used as a school for over a century, first kept by Mrs. Dennis (from about 1750), and after by Mrs. Stevenson. James Boswell's daughter Veronica was a pupil of the latter, and Mrs. Piozzi (Dr. Johnson's Mrs. Thrale) was also there when a child. In her autobiography she states that "my temporary abode was the great school in Queen Square, where Mrs. Dennis and her brother, the Admiral Sir Peter Dennis, said I was qualified, at eight years old, for teacher rather than learner, and he actually did instruct me in the rudiments of navigation, as the globes were already familiar to me." The young ladies filled one gallery in the church, to which they went in relays in a stately old coach (a distance of about 100 yards!). The coach was afterwards kept in an upper room, that the girls might practise the art of getting in and out of it in a becoming manner.

After the Zeppelin raid of September 8, 1915, hardly a whole pane of glass was left in the neighbourhood, and much brickwork was badly pitted by flying fragments. But not a soul was hurt; the bomb fell on one of the lawns and made a circular hole, which has been grassed in and retained in memory of the occasion and the providential escape of about 1,000 people sleeping that night in the Square. The base of the leaden statue in the gardens was injured slightly. It should be said in passing that this statue is not of Queen Anne, as popularly supposed, but of Queen Charlotte, wife of King George III. It is stated in a "History of the Jews' College" (which body occupies Queen Square House) that George III. lived there for a time in the charge of his physician. This and other statements in the same book are of doubtful authenticity.

Except that the locality is very quiet, and by reason of the run of the streets free from much traffic, we can advance no reason why Queen Square became a "hospital neighbourhood." Between the several founders there appears to have been no conference, and, but for the usual friendly and neighbourly relationship of the institutions, no working connection of hospital with hospital. The first hospital to arrive in the neighbourhood was the London Homœopathic Hospital, which commenced work in Great Ormond Street in 1849, and in recent years extended the building to Queen Square, an operation which caused the disappearance of four of the old houses (one—at the corner—with a bayed front). Next came the Hospital for Sick Children, which now occupies a large site fronting on Great Ormond Street, and divided by Powis Place from the Homœopathic and the back of the National. On the same site, or rather part of it, once stood Powis House, built for the second Marquis of Powis, but later used for a large part of the eighteenth century by the French and Spanish Ambassadors. There were many (and are still a few) fine houses in Great Ormond Street, which thoroughfare connects Queen Square with Lamb's Conduit Street.

The London Homœopathic Hospital.

In 1859, following the money-raising efforts of Lord Mayor David Wire and the Misses Chandler, the National Hospital for the Paralysed and Epileptic made its appearance in one small house (ten others in the Square have since been acquired). Next, in 1867, came the Alexandra Hospital for Children with Hip Disease—destined to absorb three more houses, which are shown in one of the illustrations. Except for the Italian Hospital (1884), the Alexandra was the last to come, and is the first to go, having sold the hospital buildings prior to a move to the country, first to Kettlewell House, Swanley, Kent, and then to the new hospital elsewhere when the buildings are ready.

Of course, Queen Square can make no claim to them, but within easy walking distance there are five other hospitals. There are probably more hospitals to the square mile in this neighbourhood than in any other in London.

Queen Square, Bloomsbury, 1812 (from the North).

## Figures and Tables

**Figure f1:**
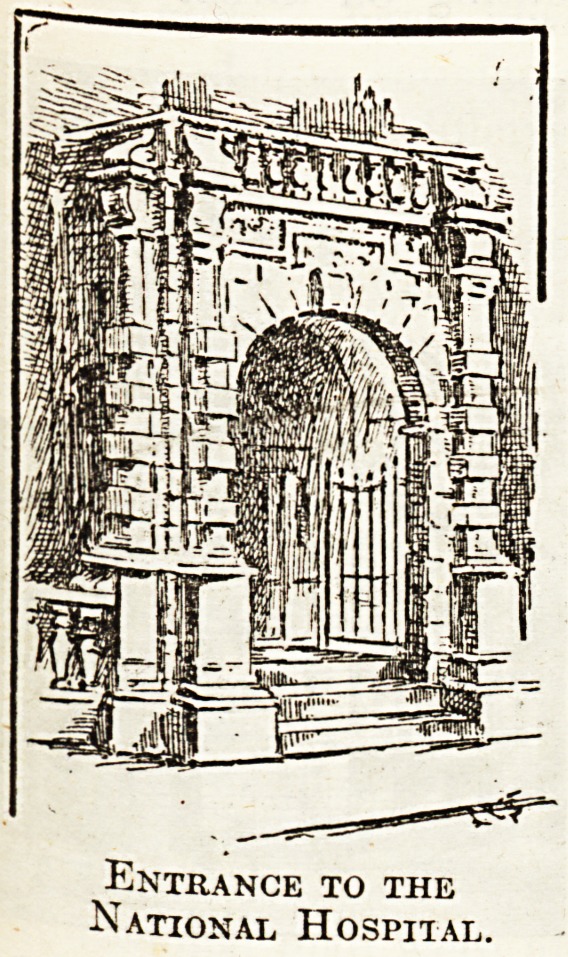


**Figure f2:**
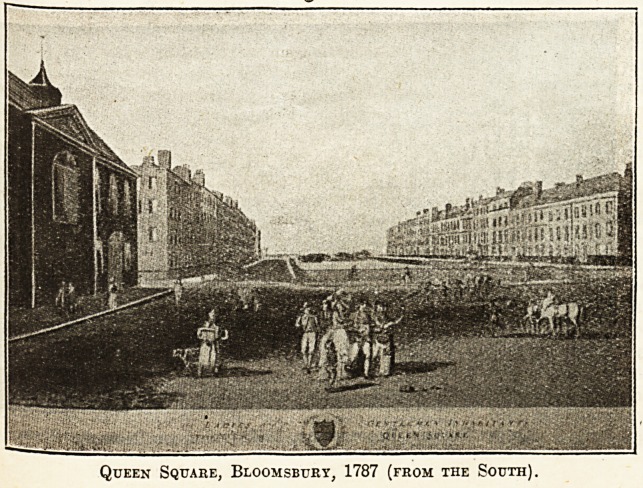


**Figure f3:**
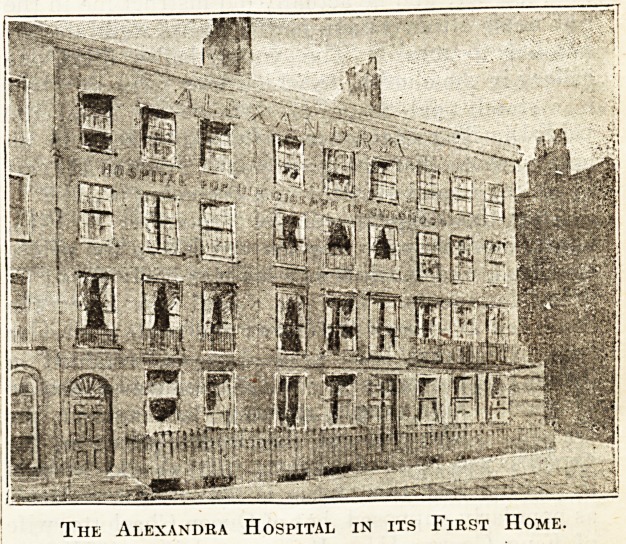


**Figure f4:**
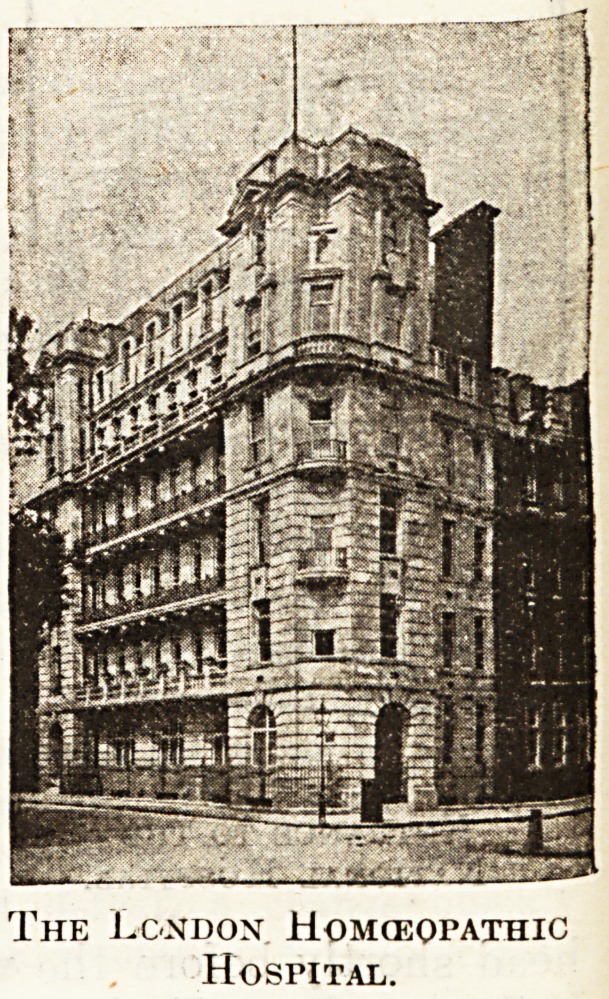


**Figure f5:**